# Selection of new sweetpotato hybrids for West Africa using accelerated breeding scheme and genotype × environment interaction under drought stress

**DOI:** 10.1038/s41598-023-33593-2

**Published:** 2023-04-20

**Authors:** Issa Zakari Mahaman Mourtala, Happiness Ogba Oselebe, Dan-jimo Baina, Ifeanyi Maxwell Nwankwo Innocent, Aristide Carlos Houdegbe, Souleyman Oumarou, Samuel C. Chukwu, Baragé Moussa

**Affiliations:** 1 Department of Natural Resources Management, National Institute of Agronomic Research of Niger, Niamey, Niger; 2 Department of Crop Production and Landscape Management, College of Agricultural and Science, Ebonyi State University, Abakaliki, Nigeria; 3 National Root Crops Research Institute, Umudike, Nigeria; 4 Faculty of Agronomic Sciences, Laboratory of Genetics, Biotechnology and Seed Science, University of Abomey-Calavi, Abomey-Calavi, Benin; 5 Faculty of Agronomy, Abdou Moumouni University of Niamey, Niamey, Niger

**Keywords:** Genetics, Plant sciences

## Abstract

West Africa is a dry region and drought tolerant sweetpotato cultivar was not reported. The objective of this study was to develop higher yielding drought tolerant sweetpotato hybrids following accelerated breeding scheme (ABS), and study G × E interaction. During advanced yield trial, the assessment of clones was conducted in six locations: four in Niger and two in Nigeria. Data were collected on storage root yield (SRY), harvest index (HI) and root dry matter content (DMC). Twenty-three hybrids were evaluated under drought and irrigation. Terminal drought was imposed. SAS and GenStat softwares were used for analyses. Based on drought susceptibility index (DSI), drought tolerant expression (DTE) and HI, clones 4 × 5 – 3, 9 × 7 – 1, 5 × 9 – 2, 3 × 6 – 2, and 3 × 12 – 3 were the best in SRY under drought stress and well-watered in combined data. Using AMMI stability value (ASV) and stability cultivar superiority (SCS), results revealed that the most superior cultivars were unstable. Clones 12 × 5 – 1 and 9 × 10 – 1 were recommended under drought for SRY stability combined with high DMC and high total carotene (TC). Under irrigation, the 13 × 8 – 2 is good candidate for stability across all locations combined with high DMC and medium TC, while clone 4 × 3 – 2, 13 × 8 – 2, 4 × 6 – 2 and 6 × 8 – 5 were stable SRY with high DMC. Therefore, these hybrids could be evaluated at on-farm trials to release the best to farmers.

## Introduction

Sweetpotato [*Ipomoea batatas* (L.) Lam] is an important root crop grown in more than 115 countries in the world^1^. Sweetpotato worldwide production of 2020 was 89,487,835 tons, with area of production 7,400,472 ha^2^. Asia is the largest (62.6%) producer, followed by Africa 32.2%^1^. In West African production data, Nigeria comes first with 3,867,871 tons, while Niger ranks fourth (209,864 tons)^2^. The rainy and dry seasons support its production in Niger, but its growth is mostly supported by the rainy season in Nigeria. In sub-Saharan Africa, sweetpotato is a major food crop renowned for its role in market value as well as in food security, nutritional and health benefits^3,5^. Generally, among other crops it ranked ninth in protein production in developing countries, seventh in digestible energy production, sixth in dry matter production, and fifth economically^6^.

Sweetpotato is particularly sensitive to water scarcity during the period of establishment including vine development and storage root initiation. Low et al.^7^ reported that the main sweetpotato production constraints constituting yield gaps, include drought, poor access to quality planting material, pests and pathogens, and poor soil fertility. In sub-sahara Africa (SSA), total rainfall during the growing period was the most yield limiting factor contributing to sweetpotato yield gaps^8^. Average SSA sweetpotato storage root yields are far below attainable yield levels with 3, 3, 7.5, and 8 t/ha in West, Southern, Central and East Africa, respectively^1^. According to^7^, today drought tolerance has been the key abiotic constraint addressed by breeding in Southern Africa, with 19 drought tolerant orange-fleshed sweetpotato (OFSP) cultivars released in Mozambique^9^, and many of them have been introduced to other African countries. Therefore, there is a need of drought tolerant sweetpotato varieties for other African regions.

Genotype performance is influenced by the environment in which it is grown^10,12^. Genotype × environment (G × E) interaction becomes of practical significance only when cross-over interactions occur^13,14^. The genotype × environment significantly affects the growth and productivity of sweetpotato, as noted when testing cultivars or advanced lines under managed drought across sites in Kenya^15^, varying eco-geography in Peru^16^, across sites and over years in South Africa^17^, under managed drought across locations in Mozambique^18^, across varying agro-ecologies in Tanzania^19^, and across locations in Indonesia^20,21^. The G × E causes difficulty to selection of clones with wide adaptation, which may delay the cultivar release^22^. Therefore, genotypes need to be tested over seasons across many environments in order to select the promising genotype for a specific environment or for stable superior performance across environments.

Statistical analysis such as AMMI^23^ and GGE biplot^10,24^ analyses are widely used in genotype by environment interaction (GEI) analysis. Stability methods were used to select superior or stable sweetpotato cultivars in Indonesia^21^, and for drought tolerance in Mozambique^7,18^, and South Africa^19^.

The overall objective of this study was to develop drought tolerant sweetpotato cultivars for farmer in West Africa. To achieve this, one method is the use of accelerated breeding scheme (ABS)^16^. The time taken to release a cultivar is significantly high (7–8 years) using traditional methods compared to 3–4 years in ABS method. Therefore, the objectives of this study are (i) to develop high yielding drought tolerant sweetpotato cultivar for farmers in West Africa through ABS, (ii) identify the magnitude of GEI in clone hybrids under drought stressed and well-watered conditions; (iii) identify the best clones under drought and well-watered conditions for storage root yield, harvest index, and dry matter content.

## Methods and materials

### Experimental site and planting material

**Figure 1:**
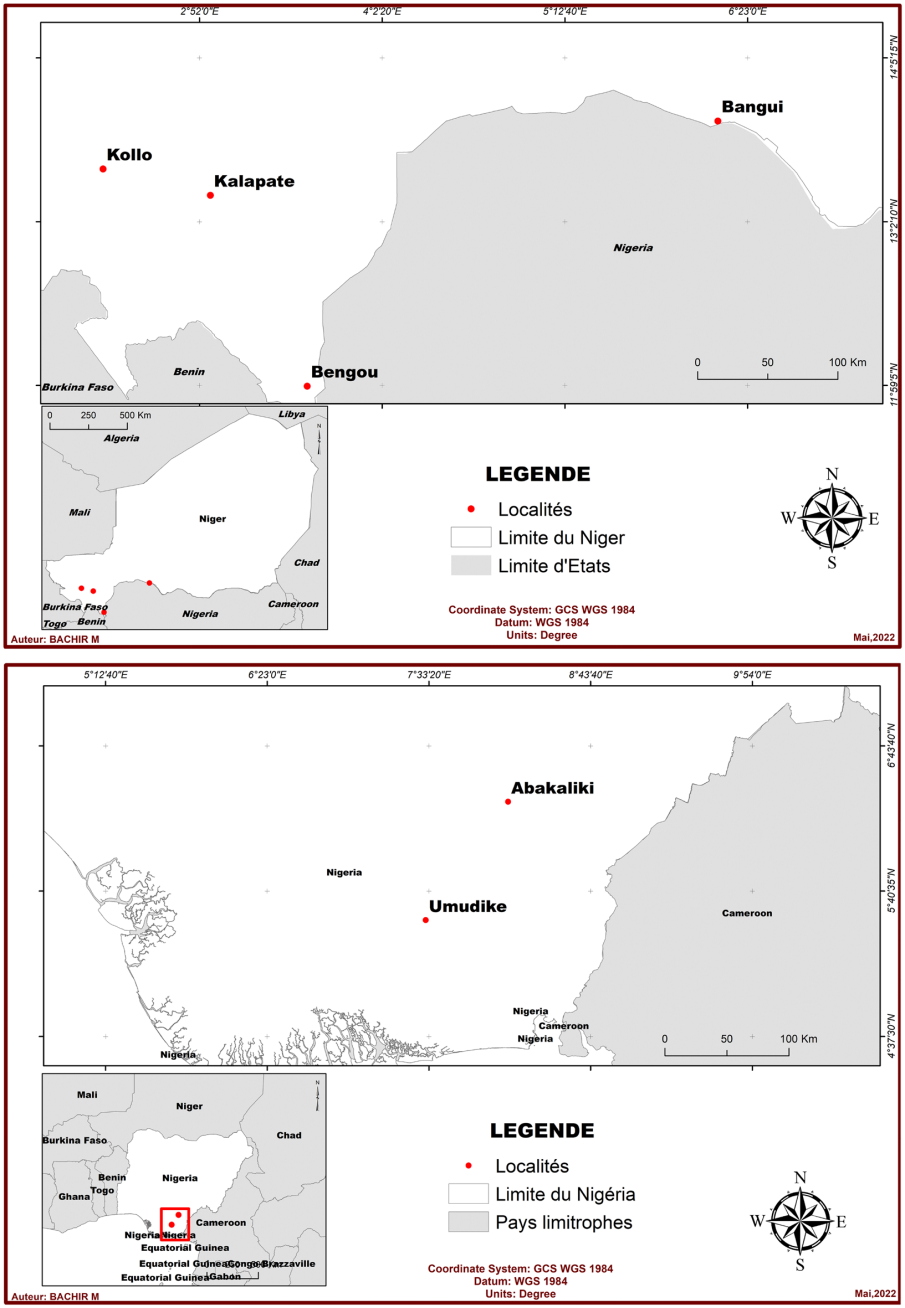
The six locations used for AYT four from Niger (above) and two from Nigeria (below).

During crossing block, 13 × 13 genotypes were crossed in diallel mating design method 3 model 1^25^ in 2019 at Ebonyi State University, Nigeria. Seedling nursery (SN) and observational trial (OT) was carried out in 2020 in Nigeria. Furthermore, preliminary yield trial (PYT) was done in Nigeria in 2021, while advanced yield trial (AYT) was carried out in Niger and Nigeria in 2021. Plant collection and used was in accordance with all the relevant guidelines such as data passports and import permit. Trials were conducted in four locations in Niger (Kollo, Kalapate, Bengou research stations under National Institute of Agronomic Research of Niger (INRAN), Bangui at farmer’s site in Niger and two sites in Nigeria (Ebonyi State University (EBSU) field experiment and National Root Crop Research Institute NRCRI Umudike) (Fig. 1). The figures were created using software QGis 3.16. Sites Niger and Nigeria were different agroecological zones. During the last ten years (2012–2021), the mean rainfall in Kollo, Kalapate, Bengou and Bangui zones of Niger were 538.80, 601.90, 916.4, and 566.90 mm respectively, while Abakaliki and Umudike have above 1929.40 mm each. Twenty-three hybrid clones selected from PYT were used as planting material (Table 1).

**Table 1:** Description of 23 progenies used in AYT.

N°	Hybrids	Codes	SC	FC	TC (ug/g)
1	AYT/2020/1 × 2 – 1	C1	Yellow	Pale orange	55.74
2	AYT/2020/1 × 7 – 6	C2	Purple	Dark yellow	54.25
3	AYT/2020/1 × 9 – 5	C3	Orange	Cream	18.46
4	AYT/2020/3 × 6 – 2	C4	Purple	Dark yellow	20.71
5	AYT/2020/3 × 12 – 3	C5	Orange	Intermediate Orange	38.55
6	AYT/2020/3 × 13 – 1	C6	Orange	Intermediate Orange	46.04
7	AYT/2020/4 × 3 – 2	C7	Cream	Cream	16.32
8	AYT/2020/4 × 5 – 3	C8	Cream	White	5.67
9	AYT/2020/4 × 6 – 2	C9	White	White	5.84
10	AYT/2020/5 × 9 – 2	C10	Red	Intermediate Orange	17.39
11	AYT/2020/5 × 12 – 1	C11	Orange	Intermediate Orange	51.84
12	AYT/2020/6 × 8 – 5	C12	White	White	6.62
13	AYT/2020/6 × 8 – 15	C13	Orange	Dark orange	63.13
14	AYT/2020/6 × 13 – 1	C14	Orange	Dark orange	71.38
15	AYT/2020/7 × 6 – 1	C15	Red	White	18.07
16	AYT/2020/8 × 6 – 1	C16	Cream	Dark cream	16.97
17	AYT/2020/9 × 7 – 1	C17	Cream	Cream	17.59
18	AYT/2020/9 × 8 – 1	C18	Orange	Pale orange	60.02
19	AYT/2020/9 × 10 – 1	C19	Red	Intermediate Orange	58.28
20	AYT/2020/9 × 13 – 2	C20	Red	Intermediate Orange	56.64
21	AYT/2020/11 × 7 – 1	C21	Purple	Dark cream	6.89
22	AYT/2020/12 × 5 – 1	C22	Yellow	Dark yellow	40.14
23	AYT/2020/13 × 8 – 2	C23	Orange	Dark cream	19.43

*AYT* advanced yield trial, *C* clone, *SC* skin colour, *FC* flesh colour, *TC* total carotene.

The soil proprieties of the six environments are presented in Table 2.

**Table 2:** Characteristics of soil of the six studied locations in Niger and Nigeria.

Parameters	Locations					
	Kollo (E1)	Kalapate (E2)	Bengou (E3)	Bangui (E4)	Abakaliki (E5)	Umudike (E6)
Soil physical parameters						
% Sand	80.4	80.4	71.4	76.4	69.4	68.4
% Silt	2.8	9.8	11.8	6.8	13.8	8.8
% Clay	16.8	9.8	16.8	16.8	16.8	22.8
Soil chemical parameters						
pH (H_2_O)	6.2	5.9	5.5	5.6	5.3	4.1
OC (%)	1.37	0.57	0.4	0.48	1.7	2.79
OM (%)	2.37	0.98	0.7	0.84	2.93	4.81
TN (%)	0.042	0.028	0.028	0.042	0.056	0.156
AvP (mg/kg)	12.6	9.3	10.7	13.2	15.8	34.5
Exchangeable cation (cmol/kg)						
Ca	4.0	2.4	3.6	3.2	6.4	2.4
Mg	2.4	1.6	2.8	2.4	3.6	1.6
Na	0.054	0.104	0.087	0.052	0.096	0.139
K	0.087	0.075	0.103	0.08	0.123	0.154

*E* environment.

### Experimental design and drought management

Split plot arranged in Alpha Lattice Design with 2 replications was used as the experimental design in the six locations. The water regime (water stress and well-watered) was main plot factor and genotype was the sub-factor. The spacing between sub-blocks, blocks and replications were 1, 1.5 and 2 m, respectively. All the trials were established from August to December 2021. Overhead irrigation was carried out for all the blocks for 8 weeks^4,26^, then terminal drought stress was imposed in two blocks while, the other two blocks were irrigated. For the well-watered (WW), irrigation was stopped one week before harvesting. Selection was based on laid down criteria^9^ i.e., clones with storage root yield (SRY) greater than the trial grand mean were selected. Fertilizer NPK 15–15–15 was applied at a rate of 6 g/stand at 4 and 8 six week after planting (WAP). First and second weeding were done at 4 and 8 WAP too. Storage roots were dug manually at 17 WAP.

### Data collection

The following data were collected: storage root yield estimated at tonnes/hectare (SRY t/ha), harvest index (%HI) and dry matter content (%DMC). Sweetpotato descriptor^27^ was used to record skin and flesh characteristics of storage root. The methods described by^28^ were followed to determine dry matter content (DMC). A sample of 50 ± 5 g was collected from the medium free of disease storage roots of each genotype and kept in a paper sampling bag. Each storage root from each sample was peeled and cut from the middle to collect the sample. These samples were dried in an oven (Gravity Convection Incubator 4EC in Niger and Electrothermal oven, model DHG in Nigeria) at 70 °C for 72 h. Dried samples were weighed with an electronic balance and then, the DMC was calculated as follows: %DMC = [(Dry weight/Fresh weight) × 100].

### Data analysis

Data on storage root yield (t/ha), harvest index (%HI) and root dry matter content (%DMC) were subjected for analysis of variance (ANOVA) across six locations under drought stress and well-watered using statistical analysis SAS program 9.3^29^. Data for drought and irrigation was analysed separately using SAS software. Then, in each water regime, data were analysed in three steps. In the first step, phenotypic data were analysed separately for each treatment in each location. In the second step, data was analysed for each treatment across locations of each country. Third step, data was analysed in each water treatment conditions across the six locations for G x E interaction. The mean differences among treatments were detected by LSD at 95% of confidence. The ANOVA showed significant G x E interaction effect, and this led to the partitioning of the genotype-by-environment interaction (GEI) component using Additive Main Effect and Multiplication Interaction (AMMI), and GGE biplot models in GenStat software 17th edition^30^. This allowed to check the adaptability and to determine the stability of the 23 hybrids evaluated across the six locations of Niger and Nigeria.

The linear model of GEI analysis using AMMI analysis by^31^ is:

1 $${\bar{{Y}}}_{{{\text{ijr}}}} = \mu + {\text{G}}_{{\text{i}}} + {\text{E}}_{{\text{j}}} + \sum\limits_{k = 1}^{m} {\lambda_{{\text{k}}} \alpha_{{{\text{ik}}}} \Upsilon_{{{\text{jk}}}} + \rho_{{{\text{ij}}}} }$$

where: ${\bar{{Y}}}_{{{\text{ijk}}}}$ = the yield of the rth replicate of ith genotype in jth environment, µ is the overall mean, G_i_ = is the main effect of the ith genotype, Ej = is the main effect of jth environment, λ_k_ = the square root of the eigen value of the kth IPCA axis, α_ik_ and ϒ_jk_ = the principal component scores for IPCA axis k of the ith genotypes and the jth environment, ρ_ij_ = the deviation from the model.

To estimate stability of the genotypes, AMMI stability value (ASV) was computed according to^32^ as follow:

2 $$\mathrm{AMMI \, stability \, value }(\mathrm{ASV}) =\sqrt{}[\frac{SSIPCA1}{SSIPCA2}\left(IPCA1\right)]2+[IPCA2]2$$

where: IPCA1 and IPCA2 were the Interaction Principal Component Analysis axes 1 and 2. They are the first and second from IPCA scores for each genotype from the AMMI analysis. SSIPCA1 and SSIPCA2 were the sum of square of IPCA 1 and 2.

Superiority measure of stability (P_i_) was computed according to the formula given by^33^ to determine superiorities among the cultivars in the studied environments. The formula was as follow:

3 $${\text{P}}_{{\text{i}}} = \, \Sigma \left( {{\text{X}}_{{{\text{ij}}}} - {\text{ M}}_{{\text{j}}} } \right)^{{2}} /{\text{2n}}$$

where: X_ij_ = the yield of the ith genotype in the jth site or environment, M_j_ = the maximum response among all progenies in the jth site or environment, n = number of site or environments.

The linear model of GGE biplot based on Singular Value Decomposition (SVD) of the principal component (PC) was given by^11^:

4 $${\bar{{Y}}}_{{{\text{ij}}}} - \mu_{i} - \beta_{j} = \sum\limits_{k = 1}^{t} {\lambda_{{\text{k}}} \alpha_{{{\text{ik}}}} \Upsilon_{{{\text{jk}}}} + \varepsilon_{{{\text{ij}}}} }$$

where: ${\bar{Y}}_{{{\text{ij}}}}$ is the yield performance in location j from genotype i, µ_i_ is the overall average, β_j_ is the main effect of location j, k is the number of principal components (PC), λ_k_ is the singular value of the kth PC, α_ik_ and ϒ_jk_ are the scores of ith genotype and jth environment respectively for PC_k_, ε_ij_ is the error of genotype i in location j.

Also, based on stress tolerance index, clones with high yield potential and high stress tolerance were identified. For each clone, based on their storage root yield under water deficit and normal irrigation, the drought tolerance indices below were computed^34–36^:

5 $${\text{Stress}}\;{\text{Susceptibility}}\;{\text{Index}},{\text{ SSI }} = \, \left[ {\left( {{1 } - \frac{Ys}{{Yp}}} \right)} \right] \, /{\text{ SII}}$$

where Stress Intensity Index, SII = $\left( {{1 } - \frac{{\overline{\Upsilon }s}}{{\overline{\Upsilon }p}}} \right)$.

6 $$\mathrm{Geometric \, Mean \, Productivity},\mathrm{ GMP }= \sqrt{Yp \times Ys}$$

7 $$\mathrm{Drought \, tolerant \, efficiency }(\mathrm{DTE\%}) =\frac{Ys}{\mathrm{Yp}} \times 100$$

where: Ys = Yield of a genotype under drought deficit, Yp = Yield of a genotype under normal irrigation, $\overline{\Upsilon }s$ = Mean yield of all genotypes under drought deficit, $\overline{\Upsilon }p$ = Mean yield of all genotypes under normal irrigation.

Regarding the physico-chemical analysis, sample was taken from each clone and analysed in duplicate for total carotene content at the National Root Crop Research Institute (NRCRI) in Nigeria (Table 1). Soil chemical and physical analysis of six locations was analysed at Ebonyi state university (EBSU) (Table 2).

## Results

### Combined ANOVA of three traits

A combined analysis of variance under drought stress and normal condition was performed for SRY, HI and DMC (Table 3). The results exhibited very highly significant (p < 0.001) difference among the six environments and the tested clones for SRY, HI and DMC under managed drought and well-watered conditions, except SRY under irrigation (p < 0.05). Besides, the results revealed very highly significant (p < 0.001) genotype x environment (G × E) interaction effects for all the traits under both conditions except SRY under irrigation where significant differences were observed (p < 0.05).

**Table 3:** Combined analysis of variance of storage root yield, harvest index and dry matter in multi-environment testing under drought stress and irrigation.

Source of variance	DF	Mean square					
		SRY (t/ha)		HI (%)		DMC (%)	
		DS	WW	DS	WW	DS	WW
Genotype (G)	22	71.79***	358.47***	613.42***	844.36***	117.80***	123.02***
Block (B)	6	15.69^ns^	10.21^ns^	317.06***	81.41^ns^	27.06**	7.09^ns^
Environment (E)	5	273.61***	204.63*	1575.62***	2096.95***	239.32***	376.83***
G x E	110	30.51***	105.11*	158.57***	205.98***	25.05***	19.20**
Error	132	10.67	110.1	73.82	91.88	9.09	11.67
CV (%)		44.84	58.08	29.36	27.14	9.55	11.63
R^2^		0.82	0.68	0.81	0.81	0.85	0.81

*Significant at 5%, **Significant at 1%, ***Significant at 0.1%, ^ns^not significant, *DF* degree of freedom, *SRY* storage root yield, *HI* harvest index, *DMC* dry matter content, *DS* drought stress, *WW* well-watered, *CV* coefficient of variation, *R*^*2*^ R-square.

### Drought parameters

The results of Table 4 showed that 14 out of 23 clones had drought susceptible index (DSI) less than 1.0, while seven had SRY greater than grand mean under drought (7.28 t/ha) and 10 clones had values higher than the grand mean under irrigation (14.88 t/ha). Genotypes 1 × 9 – 5, 6 × 8 – 5, 8 × 6 – 1 and 13 × 8 – 2 recorded DSI less than 1.0, each with respective values of: 0.92, 0.62, 0.82 and 0.84. The performance of the four clones for SRY under non irrigated condition were greater than the grand mean (7.28 t/ha) moreover, the same clones had higher means than the grand mean (14.88 t/ha) under irrigation. These genotypes also had high values for drought tolerance expression (DTE) and percent of harvest index (%HI) greater than grand mean under drought (29.26%) and under irrigation (35.31%). Twelve hybrids had HI greater than grand mean under drought (29.26%) and 11 under irrigation (35.31%). However, genotype 7 × 6 – 1 had the highest SRY under drought (13.12 t/ha) and irrigation (30.47 t/ha), but DSI was greater than 1.0. The geometric mean productivity (GMP) for the clones 4 × 5 – 3 (5.83), 9 × 7 – 1 (6.57), 5 × 9 – 2 (6.95), 3 × 6 – 2 (7.00), and 3 × 12 – 3 (7.06) were low, but their respective SRY under both conditions were lesser than grand mean. For root dry matter content (DMC), the value without irrigation was higher than with irrigation in most of the cases with the average without irrigation (31.58%) and with irrigation (29.36%). Thirteen clones had DMC greater than grand mean under drought (31.58%) and 14 under well-watered (29.36%).

**Table 4:** Storage yield root and its drought susceptible index, geometric mean productivity and drought tolerant expression as well as harvest index and root dry matter content under both water regimes.

Hybrids	SRY					%HI		DMC	
	DS (t/ha)	WW (t/ha)	DSI	GMP	DTE	DS (%)	WW (%)	DS (%)	WW (%)
AYT/2020/1 × 2 – 1	10.18	21.69	1.04	14.86	46.91	38.90	48.36	29.31	31.16
AYT/2020/1 × 7 – 6	4.58	15.42	1.38	8.41	29.73	20.59	34.75	33.86	30.79
AYT/2020/1 × 9 – 5	10.47	19.70	0.92	14.36	53.14	36.05	37.26	33.57	29.86
AYT/2020/3 × 6–2	5.14	9.54	0.90	7.00	53.85	26.63	25.96	30.98	25.24
AYT/2020/3 × 12 – 3	5.23	9.55	0.89	7.06	54.75	17.04	21.68	33.72	28.63
AYT/2020/3 × 13 – 1	6.12	10.37	0.80	7.96	59.00	24.48	34.63	32.30	30.36
AYT/2020/4 × 3 – 2	6.85	13.83	0.99	9.73	49.53	25.00	30.81	33.69	33.08
AYT/2020/4 × 5 – 3	4.50	7.56	0.79	5.83	59.55	19.94	25.37	32.82	34.19
AYT/2020/4 × 6 – 2	7.10	16.31	1.11	10.76	43.56	39.59	51.48	34.28	31.83
AYT/2020/5 × 9 – 2	5.76	8.39	0.61	6.95	68.61	30.25	29.99	31.53	31.71
AYT/2020/5 × 12 – 1	6.64	12.52	0.92	9.11	53.04	33.18	42.89	28.14	26.54
AYT/2020/6 × 8 – 5	11.27	16.53	0.62	13.64	68.16	29.05	29.04	33.02	28.79
AYT/2020/6 × 8 – 15	6.77	11.46	0.80	8.81	59.02	24.80	30.15	31.47	29.25
AYT/2020/6 × 13–1	8.76	23.91	1.24	14.48	36.64	44.26	50.22	27.06	23.05
AYT/2020/7 × 6 – 1	13.12	30.47	1.11	19.99	43.07	32.17	41.38	31.16	26.68
AYT/2020/8 × 6 – 1	10.86	18.60	0.82	14.21	58.37	32.91	38.20	26.49	26.01
AYT/2020/9 × 7 – 1	4.01	10.74	1.23	6.56	37.34	20.72	25.14	34.37	31.34
AYT/2020/9 × 8 – 1	5.53	14.67	1.22	9.01	37.72	34.71	40.27	24.95	25.90
AYT/2020/9 × 10–1	5.58	13.57	1.15	8.70	41.08	35.43	41.76	32.35	31.30
AYT/2020/9 × 13 – 2	6.98	18.50	1.22	11.36	37.73	21.39	32.41	26.20	22.93
AYT/2020/11 × 7 – 1	6.48	11.05	0.81	8.46	58.59	24.04	25.06	35.23	32.08
AYT/2020/12 × 5 – 1	6.87	12.47	0.88	9.26	55.10	30.15	36.04	36.51	32.06
AYT/2020/13 × 8 – 2	8.75	15.30	0.84	11.57	57.22	31.74	39.37	33.47	32.61
Grand mean	7.28	14.88				29.26	35.31	31.58	29.36
LSD (5%)	2.64	6.98				6.93	7.74	2.44	2.76

*AYT* advanced yield trial, *SRY* storage root yield, *HI* harvest index, *DMC* dry matter content, *DS* drought stress, *WW* well-watered, *DSI* drought susceptibility index, *GMP* geometric mean productivity, *DTE* drought tolerant expression.

### AMMI stability value, cultivar superiority, IPCA1 and IPCA2 for SRY

The results showed larger Interaction Principal Component Analysis axis 1 (IPCA1) and Interaction Principal Component Analysis axis 2 (IPCA2) under both conditions in most of the cases (Table 5). Four out of six locations had larger IPCA1 (IPCA > 1) under drought and under irrigation conditions: Kalapate (1.43 and 18.10), Bangui (− 4.81 and 15.82), Abakaliki (1.35 and 15.66) and Umudike (2.21 and 12.91). Likewise, larger values were observed in three locations for IPCA2: Kalapate (− 3.35 and − 5.75), Bengou (1.47 and 1.90) and Umudike (1.83 and 2.15). However, small values ((IPCA < 1) were observed under drought in Kollo (E1) for IPCA1 (0.51) and IPCA2 (− 0.55). The stability cultivar superiority Pi measured and ranked genotypes according to their superiority across all tested environments from the smallest to the largest. The smallest value of stability cultivar superiority indicates the highest genotype. Table 5 exhibited that the first 5 elite genotypes under drought stress were 7 × 6 – 1 (6.19), 8 × 6 – 1 (17.71), 1 × 9 – 5 (18.17), 6 × 8 – 5 (18.35) and 1 × 2 – 1 (22.44), while under irrigation 7 × 6 – 1 (65.39), 6 × 13 – 1 (120.83), 1 × 2 – 1 (139.10), 1 × 9 – 5 (189.93) and 8 × 6 – 1 (232.73). Clones 7 × 6 – 1, 8 × 6 – 1, 1 × 9 – 5 and 1 × 2 – 1 were the best hybrids under both conditions. However, genotypes 9 × 7 – 1 and 3 × 12 – 1 under drought stress and genotypes 4 × 5 – 3 and 5 × 9 – 2 under well-watered were the least. Clones 9 × 7 – 1, 3 × 12 – 3 and 3 × 13 – 1 were the lowest genotypes without irrigation, whereas 4 × 5 – 3, 5 × 9 – 2 and 3 × 13 – 1 with irrigation. Furthermore, according to AMMI stability value (ASV), clones with ASV below 1 are stable, while above 1 are unstable. All clones were sorted by stability rank. In fact, four hybrids were stable under drought including 12 × 5 – 1 (0.29), 9 × 10 – 1 (0.58), 9 × 13 – 2 (0.59) and 5 × 9 – 2 (0.79), whereas six under irrigation 4 × 3 – 2 (0.44), 13 × 8 – 2 (0.50), 5 × 12 – 1 (0.73), 4 × 6 – 2 (0.74), 6 × 8 – 5 (0.85), and 11 × 7 – 1 (0.94) (Table 5).

**Table 5:** Storage root yield, stability cultivar superiority and stability parameters of sweetpotato hybrids under drought and well-watered conditions.

Code	Hybrid	SRY (t/ha)		IPCA1		IPCA2		SCS (Rank)		ASV (Rank)	
		DS	WW	DS	WW	DS	WW	DS	WW	DS	WW
C1	AYT/2020/1 × 2 – 1	10.18	21.69	− 1.62	− 2.82	0.50	0.33	22.44 (5)	139.10 (3)	2.95 (18)	4.57 (22)
C2	AYT/2020/1 × 7 – 6	4.58	15.42	0.66	0.98	0.43	− 1.26	83.46 (19)	352.64 (10)	1.27 (7)	2.02 (13)
C3	AYT/2020/1 × 9 – 5	10.47	19.70	− 2.34	− 1.20	0.20	− 1.08	18.17 (3)	189.93 (4)	4.20 (22)	2.22 (15)
C4	AYT/2020/3 × 6 – 2	5.14	9.54	1.04	1.05	0.75	0.15	82.14 (18)	512.34 (18)	2.01 (15)	1.70 (11)
C5	AYT/2020/3 × 12 – 3	5.23	9.55	1.32	1.38	0.69	1.41	85.30 (22)	784.74 (20)	2.47 (16)	2.65 (16)
C6	AYT/2020/3 × 13 – 1	6.12	10.37	1.31	2.23	2.07	0.00	84.82 (21)	545.24 (21)	3.13 (20)	3.61 (21)
C7	AYT/2020/4 × 3 – 2	6.85	13.83	-0.83	0.27	0.32	− 0.03	47.73 (8)	375.36 (12)	1.52 (10)	0.44 (1)
C8	AYT/2020/4 × 5 – 3	4.50	7.56	0.61	0.83	0.73	0.72	83.67 (20)	559.01 (23)	1.32 (8)	1.53 (10)
C9	AYT/2020/4 × 6 – 2	7.10	16.31	0.62	− 0.46	− 1.60	− 0.03	55.89 (11)	284.94 (6)	1.95 (14)	0.74 (4)
C10	AYT/2020/5 × 9 – 2	5.76	8.39	0.39	0.80	0.35	1.30	66.79 (15)	545.80 (22)	0.79 (4)	1.83 (12)
C11	AYT/2020/5 × 12 – 1	6.64	12.52	1.70	0.43	− 0.64	0.22	68.20 (17)	404.11 (14)	3.12 (19)	0.73 (3)
C12	AYT/2020/6 × 8 – 5	11.27	16.53	− 2.77	− 0.53	1.01	0.99	18.34 (4)	291.29 (7)	5.06 (23)	0.85 (5)
C13	AYT/2020/6 × 8 – 15	6.77	11.46	0.88	0.92	0.15	0.18	64.75 (13)	452.97 (17)	1.59 (11)	1.49 (9)
C14	AYT/2020/6 × 13 – 1	8.76	23.91	1.02	− 5.10	− 1.81	0.53	47.72 (7)	120.83 (2)	2.58 (17)	8.27 (23)
C15	AYT/2020/7 × 6 – 1	13.12	30.47	− 1.66	− 1.73	− 1.38	− 1.88	6.19 (1)	65.39 (1)	3.29 (21)	3.37 (19)
C16	AYT/2020/8 × 6 – 1	10.86	18.60	− 0.92	− 1.04	− 0.61	1.15	17.71 (2)	232.73 (5)	1.76 (13)	2.04 (14)
C17	AYT/2020/9 × 7 – 1	4.01	10.74	0.70	1.49	0.53	2.16	91.10 (23)	534.80 (19)	1.37 (9)	3.24 (18)
C18	AYT/2020/9 × 8 – 1	5.53	14.67	0.83	− 0.15	− 0.92	− 1.43	72.94 (16)	360.00 (11)	1.75 (12)	1.46 (8)
C19	AYT/2020/9 × 10 – 1	5.58	13.57	0.05	1.50	0.57	− 1.74	65.87 (14)	430.60 (15)	0.58 (2)	2.99 (17)
C20	AYT/2020/9 × 13–2	6.98	18.50	0.28	0.91	− 0.32	-3.12	56.66 (12)	296.34 (8)	0.59 (3)	3.45 (20)
C21	AYT/2020/11 × 7 – 1	6.48	11.05	− 0.65	0.45	− 0.14	0.61	49.36 (9)	448.13 (16)	1.18 (6)	0.94 (6)
C22	AYT/2020/12 × 5 – 1	6.87	12.47	− 0.09	− 0.16	− 0.23	1.32	49.40 (10)	395.95 (13)	0.29 (1)	1.35 (7)
C23	AYT/2020/13 × 8 – 2	8.75	15.30	− 0.54	− 0.05	− 0.65	− 0.50	29.98 (6)	315.06 (9)	1.16 (5)	0.50 (2)
	Kollo (E1)	3.99	12.38	0.51	1.87	− 0.55	− 4.46				
	Kalapate (E2)	6.80	18.10	1.43	− 5.73	− 3.35	− 5.73				
	Bengou (E3)	8.42	14.38	− 0.68	1.89	1.67	1.90				
	Bangui (E4)	11.07	15.82	− 4.81	− 2.58	− 0.26	− 2.58				
	Abakaliki (E5)	7.81	15.66	1.35	2.39	0.66	2.39				
	Umudike (E6)	5.60	12.91	2.21	2.15	1.83	2.15				

*C* clone, *AYT* advanced yield trial, *SRY* storage root yield, *IPCA1* interaction principal component analysis 1, *IPCA2* interaction principal component analysis 2, *SCS* stability cultivar superiority, *ASV* AMMI stability value, *DS* drought stress, *WW* well-watered.

### GGE biplot for storage root yield, harvest index and root dry matter content

#### Storage root yield (SRY)

**Figure 2:**
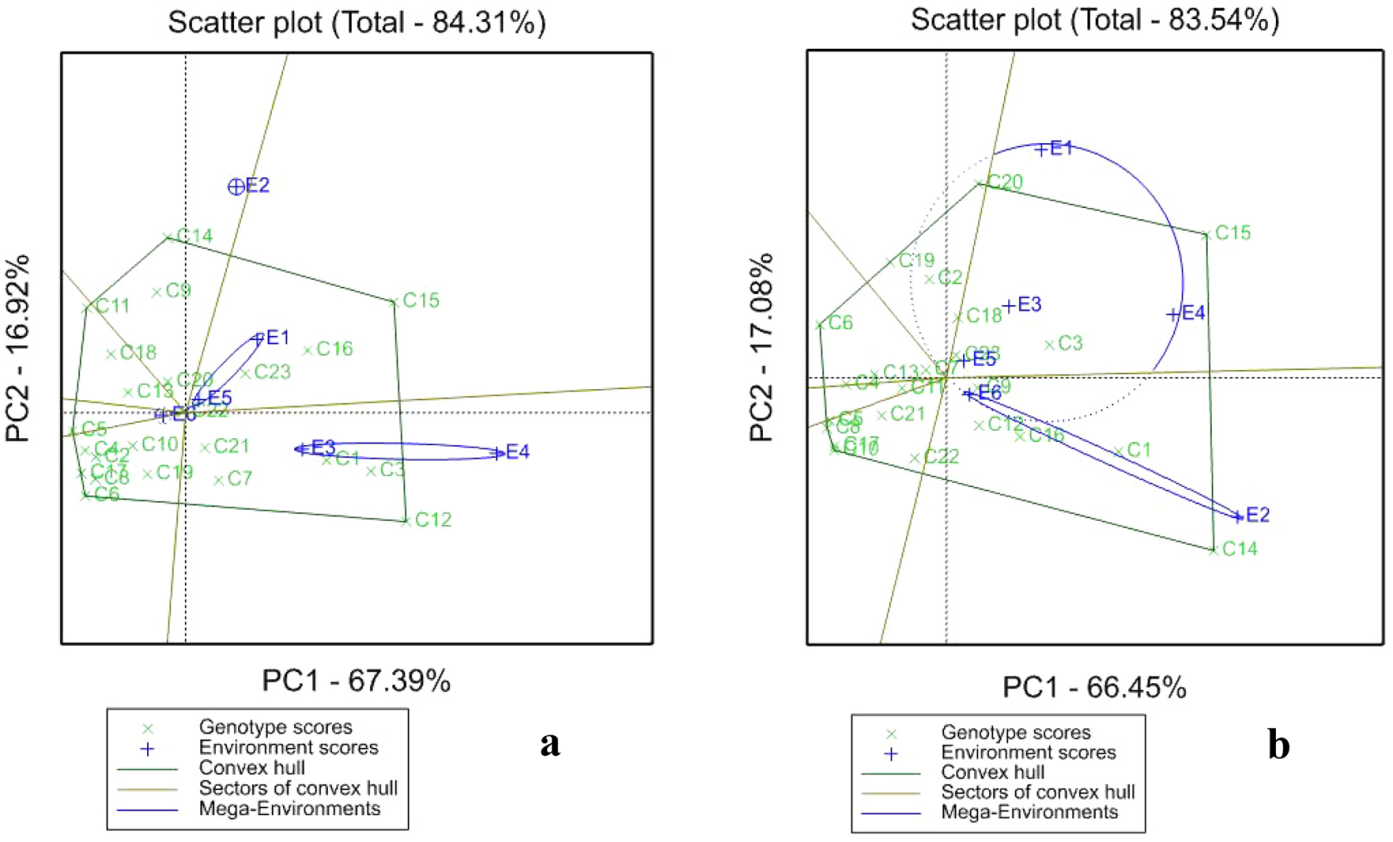
Outstanding performances of the sweetpotato clones for storage root yield in the different environments (“which won where”) and the delineated mega environments under drought (**a**) and under well-watered (**b**) conditions.

GGE biplot for storage root yield (SRY) under drought and irrigation are presented in Fig. 2. The “which-won-where” for SRY (Fig. 2) showed that the first two principal components axes (PCs) explained 84.31% of total variation under drought (Fig. 2a) and 83.54% under irrigation (Fig. 2b). Genotypes to the left of the line that passe through the biplot origin line are with below average value, whereas genotypes to the right are with above average values of the interest traits The GGE biplot under drought showed that clones C15 (7 × 6 – 1) and C12 (6 × 8 – 5) had high yield and adaptability for E5, E1, E3 and E4, since they were located at the vertices of the polygons, while C1 (1 × 2 – 1), C3 (1 × 9 – 5) and C16 (8 × 6 – 1) had high yield but with low adaptability at E5, E1, and E3. Under irrigation clones C14 (6 × 13 – 1) and C15 (7 × 6 – 1) had high SRY and widely stable at E3, E4 and E2. Under drought C15 performed well at E1 and E5, while, C12 performed better at E3 and E4. Under irrigation C20 was good at three environments (E1, E3 and E5) as well as C15 (E4, E3 and E5), while C14 performed better at E2 and E6.

Three mega-environments were identified by GGE biplot under drought stress, in which E2 constitute the first mega-environment, E1, E5 and E6 the second and E3 and E4 the third mega-environment. Under irrigation, two mega-environments were formed with E1, E3, E4 and E5 as first mega-environment and E2 and E6 as the second mega-environment. In this study, under drought, E4 (Bangui) provided the highest SRY of 10.07 t/ha followed by E3 (Bengou) 8.42 t/ha and under irrigation E2 (Kalapate) had the highest SRY of 18.10 t/ha following by E4 (Bangui) 15.82 t/ha. E1 (Kollo) was the poor environment under drought (3.99 t/ha) and irrigation (12.38 t/ha).

#### Harvest index (%HI)

**Figure 3:**
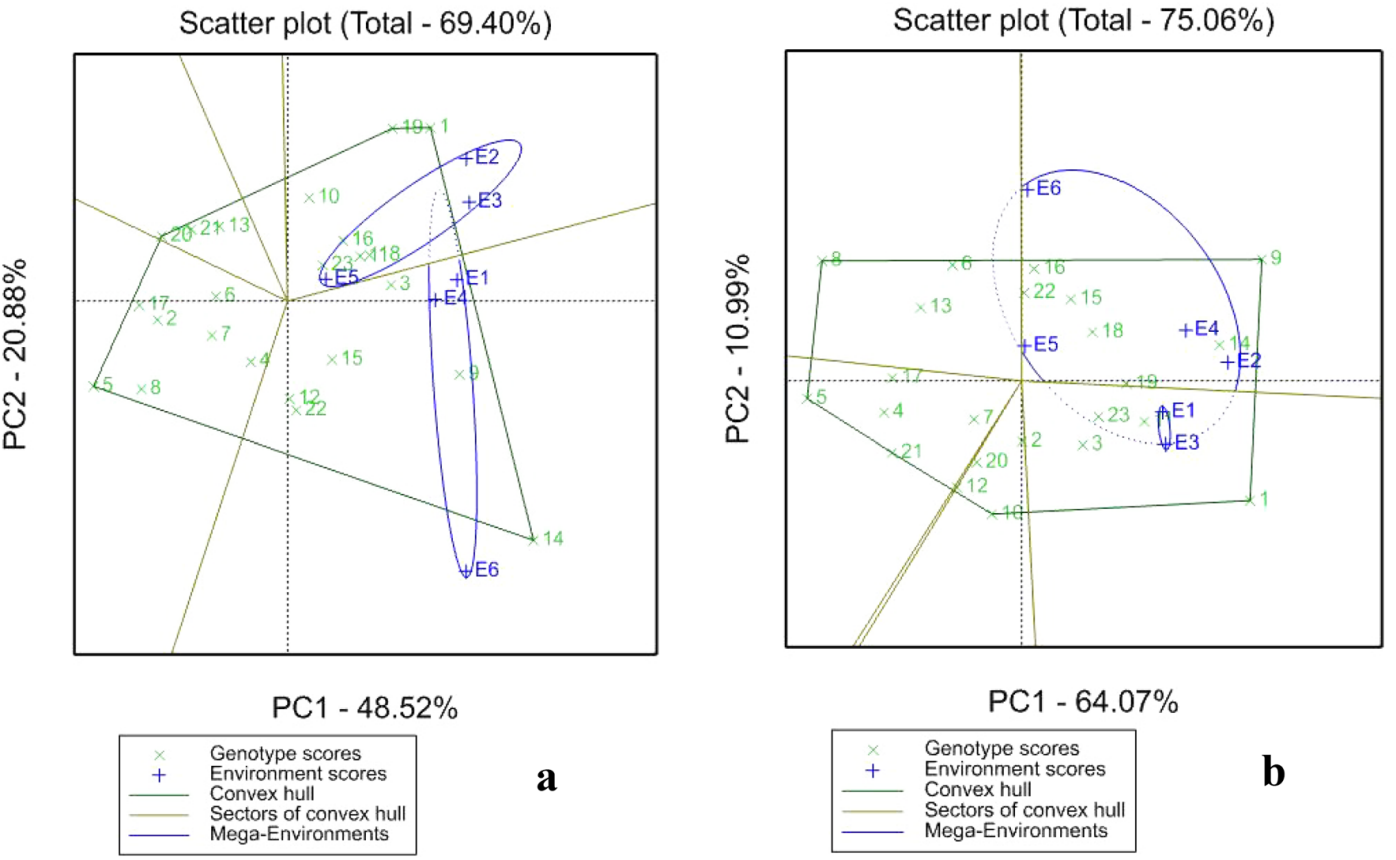
Outstanding performances of the sweetpotato clones for harvest index in the different environments (“which won where”) and the delineated mega environments under drought (**a**) and under well-watered (**b**) conditions.

Figure 3 presents the GGE biplots showing “which won where” or which clones are best for which location for harvest index (%HI). Under drought condition PCs 1 and 2 explained 48.52% and 20.88% % of the total variation, while under irrigation the two PCs PC1 64.07% and PC2 10.99%, respectively. Clones C14, C10, C9, and C1 were found by GGE biplot as the highest HI (greater than the overall average of 29.26%) and most stable genotypes at E1, E2, E3, and E4 respectively under drought condition, while clones C5, C17, C20, and C2 had HI less than the average. Under well-watered condition clones C9, C14, and C1 were identified by GGE Biplot as the promising and stable clones across all the four locations of Niger with HI above the overall average (31.35%), while C8, C6, C5, C21, and C10 registered the HI below the average.

#### Root dry matter content (%DMC)

**Figure 4:**
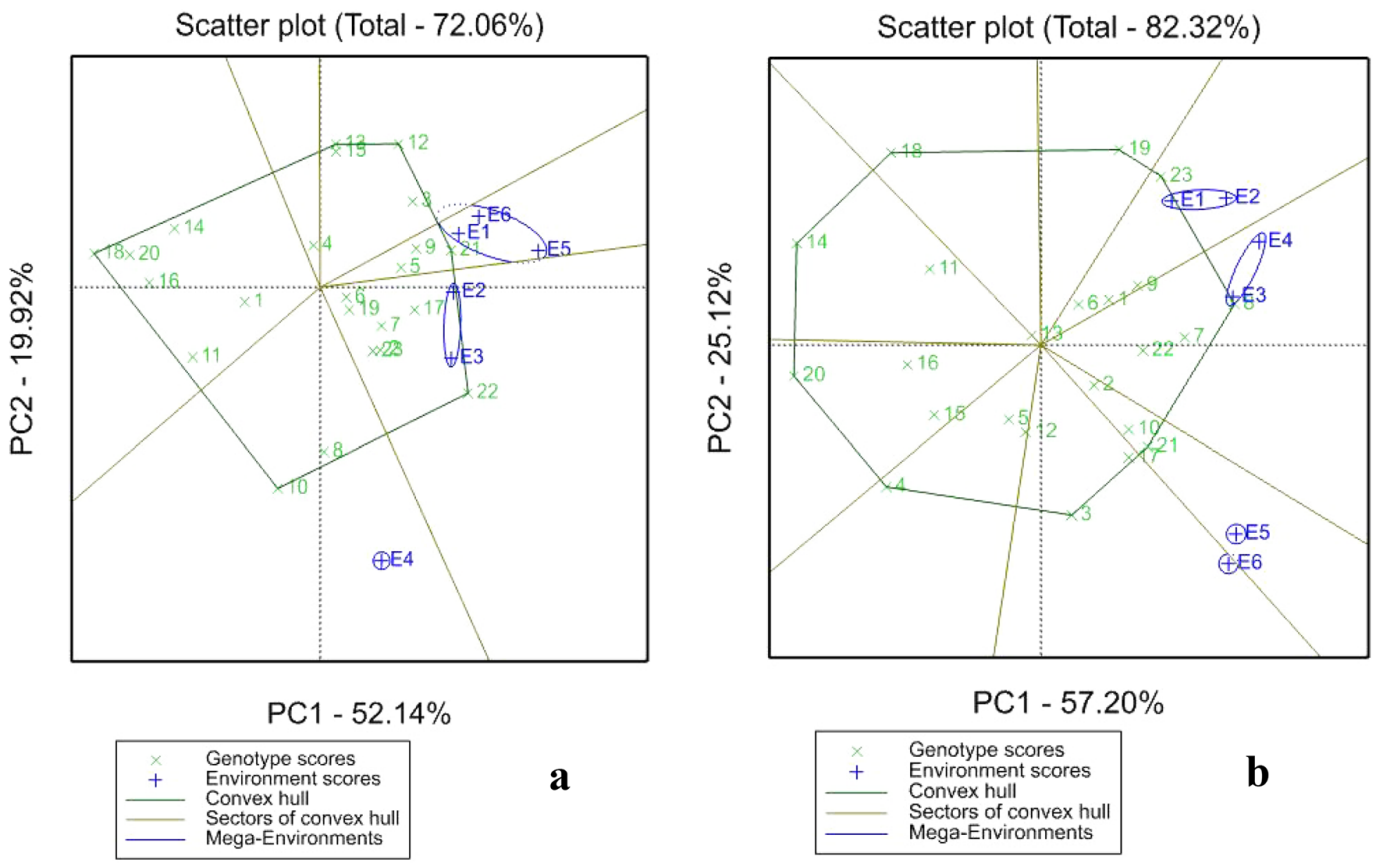
GGE Biplot showing genotypes performance according to their respective environments under drought (**a**) and irrigation (**b**) for root dry matter content.

Results of GGE Biplot of 23 hybrid clones across the six locations in Niger and Nigeria for root dry matter content (DMC) is presented in Fig. 4. The study revealed that the two PCs under drought accounted 72.06% of the total variation (Fig. 4a), while under optimum condition of good watering, 82.32% was explained (Fig. 4b). Clones C22, C17, C21, C9, C3, C12, and C5 performed well under drought, and had DMC above the overall average of 31.58%, while C18, C20, C16 and C14 had low DMC. Besides, clones C3, C21, C9, C5 and C17 performed well in four locations; include two in Niger E1 (Kollo) and E2 (Kalapaté), and two in Nigeria E5 (Abakaliki) and E6 (Umudike). Under optimum condition, PC1 (57.20%) and PC2 (25.12%) explained 82.32% of total variation. Hybrids C23, C8, C9, C7, C22, C10, and C21 had DMC above the overall average of 29.36%, while C18, C14, C20 and C4 had DMC below the average. Three mega-environments were formed under irrigation (Fig. 4b) in which first (E1 and E2), and second (E3 and E4) mega-environments constituted by Niger locations, and the third by Nigeria locations (E5 and E6). Clones gave high DMC under irrigation in all Niger locations, while Nigeria locations were the highest under drought.

## Discussions

### Combined ANOVA

The combined analysis of variance of SRY, HI and DMC across environments, genotypes, and genotypes by environments interaction indicated that the SRY, HI and DMC under both drought stress and well-watered condition were highly affected by environments. The highly significant (p < 0.001) difference among the tested genotypes for the three characters under drought stress (DS) and well-watered (WW) indicates the genetic causes of variation. The highly significant (p < 0.001) difference which existed between the six environments suggests significant differences among the environments. Genotypes’ performance was influenced by genetic constitution and the environment where they were grown^10,12^. The inconsistencies in the performances of the genotypes resulted in cross-over interaction (a difference in ranking of a genotype from one environment to another). This suggests that some genotypes were sensitive and not stable within treatments and from one environment to another. Similar results were reported on SRY and DMC by^16,18,19,37^. In Niger, Nigeria and other West African countries, rainfall is irregular and this makes breeding for drought a complex endeavour. G × E causes difficulty in selection of genotypes with wide adaptation, which may delay the cultivar release^22^. Drought can be random, periodic, or permanent, occurring late, early, or in the middle of the crop season. In this study terminal drought was imposed at advanced yield trial in ABS, which started at the onset of root initiation. In sweetpotato breeding programs, knowledge on structure of G x E is therefore crucial to facilitate recommendations of genotypes for cultivar release, and to make informed choices regarding selection of cultivars with specific or wide adaptation^16^.

### Drought parameters

In this study, DSI, DTE and HI, exhibited that clones 4 × 5 – 3, 9 × 7 – 1, 5 × 9 – 2, 3 × 6 – 2, and 3 × 12 – 3 were the best in high SRY under the two moisture-management conditions across all the six locations in Niger and Nigeria. The results for these five genotypes, showed that their DSI were less than 1.0 with high DTE, and high HI. Genotypes with DSI below 1.0 were drought tolerant cultivars, and have stable yield in both moisture-managed conditions, while the genotypes with DSI above 1.0 are considered to be highly susceptible to drought with poor yield stability^4,34^. Andrade et al.^18^ recorded the highest DTE (96.22%) and highest %HI under drought (53.52%), and under irrigation (47.07%). Sweetpotato breeding programs have significantly improved SRY production due to improved harvest index^18^. Genotypes with high GMP are desired, since it indicates that the difference between the two treatments are small. However, in this study, clones that gave high GMP had high SRY in both moisture-managed conditions. This result corroborated with the finding of^18^ who reported high yield with high GMP in two stable genotypes. Another worker^4^ reported strong and positive correlation (r = 0.94 under drought and r = 0.88 under irrigation) between GMP and SRY. Therefore, DSI, DTE GMP, and HI are good estimates for selection of high yielding tolerant sweetpotato genotypes under drought condition. Breeders can focus on these parameters for better selection in sweetpotato cultivar under drought. Genotypes with high DMC are good for storage, and could be candidate for industrial utilization. Two orange fleshed South American varieties sweetpotato had DMC of 33.00% and 37.20% respectively^38^.

### AMMI stability value, stability cultivar superiority, IPCA1 and IPCA2 for SRY

AMMI analysis results, exhibited high values under drought stress and well-watered for IPCA1 at Umudike, Kalapate, Abakaliki and Bangui and for IPCA2 at Umudike, Bengou and Kalapaté. This indicates that the clones evaluated at Umudike (E6), Bengou (E3), Kalapate (E2) and Bangui (E4) had high storage root yield (SRY) and was a result of their superior genetic performance of each genotype due to small effect of the environment. de Oliveira et al.^39^ reported larger IPCA1 in one environment out of three in their study. The higher the positive or negative IPCA value, the higher, the adaptability of a particular genotype to a specific environment^20,40^. Low values of IPCA1 and IPCA2 under drought at Kollo (E1) indicates that the environment had more influence on the clones than the genetic effects. This is in consonance with other reports^21,39^. Genotypes with low value of stability cultivar superiority (Pi) are considered the best stable clones^33^. Clones 7 × 6 – 1, 8 × 6 – 1, 1 × 9 – 5 and 1 × 2 – 1 were the highest yielding hybrids under drought and well-watered conditions in E1, E3, E4, and E5. These genotypes were found to be less affected by the environmental factors, therefore, they could be proposed for high storage root yield production across these locations. The highest genotype in particular environment is the more unstable. Unstable genotypes were influenced by environment; thus, they may be proposed for storage yield production under specific condition for a particular location. In this study, AMMI stability value (ASV) was used to check the stability of the hybrids across Niger and Nigeria agro-ecologies. The smaller the ASV the more stable the genotype across locations, but a larger value for ASV in a location indicates better adaptation of the genotype to a specific environment^41,42^. Maulana et al.^43^ used ASV and genotype stability index (GSI) to rank the stability of 23 genotypes evaluated in three locations. The ASV has also been used in Indonesia to select more stable sweetpotato^20,21^. In this study, when coupling ASV and Pi; in this study, genotypes that were ranked as highest yielder Pi were not stable according to ASV. Therefore, there is a challenge for ranking high and stable genotypes across the six studied locations of Niger and Nigeria. High and stable yielding genotypes are ideal to both farmers and plant breeders^44,45^.

### GGE biplot for SRY, HI and DMC

#### Storage root yield

Widely and narrow adapted clones were reported in this study without and with irrigation across six tested locations in Niger and Nigeria. Each category has its advantages. In fact, stable clone may adapt to many locations, while the narrowly adaptable high yielders could be better suited to particular/ specific locations. According to^46^ cultivars with adaptation to a particular environment (i.e., responsive and unstable) have that advantage to respond to environmental changes compared to widely adapted (i.e., non-responsive and stable). Locations in different mega-environment showed that clones located in the locations belonging to the same mega-environment have similar yields. Mega-environments displayed various high yielding genotypes, and this indicates the presence of cross-over G × E interaction and inconsistent performance of the tested genotypes over the environments^31^.

#### Harvest index

Clones with high percentage of harvest index (%HI) and are stable under drought and irrigation could be proposed to the breeding program. In this study, clones C14, C10, C9, and C1 could be proposed in Niger as stable genotypes for HI under drought while clones C9, C14 and C1 under irrigation. SRY and HI had the same correlation profiles with their trait stability^4^, suggesting that the indirect selection for SRY and its stability is possible through selecting simultaneously high HI with low environmental variance. Clones to the left of the line have below average values of the interest traits^24^; therefore C5, C17, C20, and C2 under drought, and clones C8, C6, C5, C21, and C10 under irrigation could be proposed under specific locations.

#### Root dry matter content

GGE biplot for root dry matter content (DMC) revealed that certain genotypes had large, and positive PC1 scores either without or with irrigation. Discrimination between genotypes is better in environments with large PC1 scores, while those with PC2 scores near zero are more representative of an average environment^47^. Accordingly, clones C3, C21, C9, C5 and C17 performed well at E1 (Kollo), E2 (Kalapaté), E5 (Abakaliki) and E6 (Umudike) under drought, whereas under irrigation C9, C1, C6, C8, and C23 were the best genotypes in Niger and C10, C21, C17, and C3 in Nigeria. These genotypes could be selected for suitability to particular locations for high dry matter content they are also good candidates for industrial purposes. Genotypes with raw DMC > 35% are suitable products in processing industry^48^. Therefore, breeding for high DMC, could be a focus of sweetpotato plant breeder. In comparison, with other studied traits (SRY and %HI), DMC was widely adapted to most of the locations. This confirmed findings of^16^ who stated that DMC is less influenced by the environment.

## Conclusion

The current study was conducted following the accelerated breeding scheme (ABS) method in order to develop promising drought tolerant cultivar within a short time. The scheme started in 2019 with 570 true seeds from 13 × 13 diallel crosses. The current stage is AYT with 23 assessed genotypes. These genotypes were evaluated under drought and irrigation across six locations in Niger and Nigeria to check their performances and the stability. As results of AYT, four hybrids were the most stable for storage root yield across all locations under drought, they include: 12 × 5 – 1, 9 × 10 – 1, 9 × 13 – 2 and 5 × 9 – 2, however, under irrigation, six were identified: 4 × 3 – 2, 13 × 8 – 2, 5 × 12 – 1, 4 × 6 – 2, 6 × 8 – 5, and 11 × 7 – 1. Clones 12 × 5 – 1 and 9 × 10 – 1 under drought had DMC above average (31.38%) with high TC (above 40 ug/g). Clone 4 × 3 – 2, 13 × 8 – 2, 4 × 6 – 2 and 6 × 8 – 5 under irrigation had DMC above average (29.36%). Under irrigation 13 × 8 – 2 had high DMC and medium TC (20 ug/g) but 4 × 3 – 2 with the highest SRY had the lowest TC, meanwhile, 5 × 12 – 1 is stable with high TC but low DMC.

Therefore from this study, clones 12 × 5 – 1 and 9 × 10 – 1 are recommended under drought for high SRY stability combined with high DMC and high TC. Under irrigation 13 × 8 – 2 is a good candidate for high DMC stability across all locations with medium TC, 4 × 3 – 2, 13 × 8 – 2, 4 × 6 – 2 and 6 × 8 – 5 are recommended for SRY stability with high DMC, 5 × 12 – 1 for SRY stability with high TC, and finally 4 × 3 – 2 for SRY stability. Furthermore, considering individual location, under drought, 7 × 6 – 1, 8 × 6 – 1 and 13 × 8 – 2 are respective good candidates for SRY at E1 (Kollo), E5 (Abakaliki) and E6 (Umudike). Moreover, 6 × 13 – 1 is best at E2 (Kalapaté) and 6 × 8 – 5, 1 × 9 – 5 and 1 × 2 – 1 at E3 (Bengou) and E4 (Bangui). Under irrigation genotypes 9 × 13–2 is recommended for E1 (Kollo), 7 × 6 – 1 for E4 (Bangui), 6 × 13 – 1 and 1 × 2 – 1 for E2 (Kalapate), 9 × 8–1 for E3 (Bengou), 13 × 8 – 2 for E5 (Abakaliki), and 4 × 6–2 and 6 × 8 – 5 for E6 (Umudike).

To complete the ABS method, these selected genotypes could be evaluated at on-farm trials for stability and for sensitiveness according to the response of their respective environments. Breeder should consider the challenges for selecting not only cultivar with high stability and high SRY, but also candidate with the attributes of high SRY, high DMC and high TC.

## Data Availability

The datasets of the current study can be requested from corresponding author with strong reason.
